# Physicochemical and Sensory Profiling of Carob Flour and Effects of Its Incorporation on Quality Characteristics of Wheat-Based Biscuits

**DOI:** 10.3390/foods15162785

**Published:** 2026-08-08

**Authors:** Fatima Zohra Makhlouf, Jesús García Zorrilla, Maria Smati, Amina Bramki, José M. Igartuburu, Antonella Pasqualone, Giacomo Squeo, Francesco Caponio

**Affiliations:** 1 Higher National School of Biotechnology Taoufik Khaznadar, Nouveau Pôle Universitaire Ali Mendjeli, BP. E66, Constantine 25100, Algeria; f.makhlouf@ensbiotech.edu.dz (F.Z.M.); m.smati@ensbiotech.edu.dz (M.S.); a.bramki@ensbiotech.edu.dz (A.B.); 2 Food Sciences Laboratory, Formulation Innovation Valorization and Artificial Intelligence (SAFIVIA), INATAA, Frères Mentouri University 1, Constantine 25000, Algeria; 3 Allelopathy Group, Department of Organic Chemistry, Facultad de Ciencias, Institute of Biomolecules (INBIO), University of Cadiz, 11510 Puerto Real, Spain; josemanuel.igartuburu@uca.es; 4 Dipartimento di Scienze del Suolo Della Pianta e Degli Alimenti (Di.S.S.P.A.), Università degli Studi di Bari Aldo Moro, Via Amendola 165/a, 70126 Bari, Italy; antonella.pasqualone@uniba.it (A.P.); francesco.caponio@uniba.it (F.C.)

**Keywords:** *Ceratonia siliqua* L., phenolic compounds, volatile profile, antioxidant activity, wheat-based biscuits, sensory evaluation

## Abstract

This study evaluated carob (*Ceratonia siliqua* L.) flour as a functional ingredient for improving the nutritional and sensory properties of wheat-based biscuits. Mature carob pods collected from eastern Algeria were processed into fine flour and characterized for physicochemical properties, antioxidant activity, and volatile and phenolic profiles. GC–MS analysis revealed a complex volatile profile dominated by organic acids, aldehydes such as furfural and benzaldehyde, and Maillard-derived compounds, including 2-acetylpyrrole and furfuryl alcohol, associated with caramel and cocoa-like aromas. LC-MS profiling identified several flavonoids and phenolic acid derivatives, including quercetin-, myricetin-, and kaempferol-glycosides, as well as gallic acid glucoside, confirming the high antioxidant potential of carob flour. Biscuits were formulated by substituting wheat flour with 30% and 60% carob flour. Sensory evaluation revealed significant increases in color intensity, caramel odor, sweetness, and chocolate-like flavor (*p* ≤ 0.05), with the 30% substitution achieving the highest overall acceptability. These sensory attributes were associated with the volatile and phenolic profiles, particularly the presence of Maillard-derived furans and flavonoid compounds. These findings demonstrate that carob flour can enhance the sensory characteristics and contribute to the functional potential of biscuits, supporting its use as a natural cocoa substitute and source of bioactive compounds in bakery products.

## 1. Introduction

Carob (*Ceratonia siliqua* L.), a resilient leguminous tree native to the Mediterranean basin, has garnered increasing scientific interest due to its diverse applications in food, agriculture, and pharmacology [1]. Traditionally used as a food source for both humans and livestock, carob has recently been valorized in human diets through the development of health-promoting food products derived from its various components, namely the pod, pulp, and seeds. In particular, the food industry has shown growing interest in carob-derived ingredients such as carob pulp flour, carob gum (locust bean gum), and carob syrup [2,3].

Carob flour, obtained from the dried pod, is naturally sweet, free from caffeine and theobromine, and widely used as an alternative to cocoa and refined sugars in bakery products and beverages [4]. Nutritionally, it is rich in dietary fiber and natural sugars, low in fat, and a rich source of essential minerals [3]. Moreover, carob flour contains significant levels of bioactive compounds, including polyphenols, flavonoids, and tannins, which contribute to its antioxidant potential and functional properties [5].

In response to growing consumer demand for functional foods that provide health benefits beyond basic nutrition [6], carob flour has emerged as a promising ingredient. Its phytochemical composition supports several documented health benefits, while its favorable sensory and technological properties make it attractive for food formulation [7]. Furthermore, it has shown potential as a functional ingredient in gluten-free bakery applications, where it contributes to improved texture, volume, and overall sensory quality due to the hydrocolloid properties of its gum (galactomannan) [4,8,9].

Despite these advantages, the incorporation of carob flour into bakery products presents several technological challenges, particularly at high substitution levels. Owing to its high dietary fiber content and the absence of gluten-forming proteins, carob flour may modify dough hydration, weaken gluten network development, and affect the structural characteristics of wheat-based products [2,4,8,10].

In addition, its naturally dark color and phenolic composition may influence consumer perception, with potential positive effects on appearance and aroma but possible negative effects related to excessive bitterness or astringency depending on the formulation. These variable outcomes reported in the literature are likely related to differences in carob cultivar, roasting conditions, particle size, substitution level, and product type, highlighting the need for a more comprehensive understanding of the technological behavior of carob flour in wheat-based systems.

The fortification of bakery and confectionery products with carob flour has been investigated in several formulations, including biscuits, cakes, muffins, and gluten-free products [8,10]. However, previous studies have mainly focused on gluten-free applications or moderate substitution levels, whereas limited information is available regarding the technological performance and sensory acceptability of wheat-based biscuits containing high proportions of carob flour. Moreover, the relationships between the chemical composition of roasted carob flour, particularly its phenolic and volatile profiles, and the nutritional, technological, and sensory characteristics of wheat-based biscuits remain insufficiently understood. This research gap is especially relevant in Mediterranean regions, including Algeria, where integrating carob into local biscuit formulations could offer nutritional, sensory, and economic advantages while promoting the use of underutilized agro-resources.

Therefore, the present study aims to evaluate the effects of high-level carob flour substitution in wheat-based biscuits on the nutritional, physicochemical, functional, technological, and sensory properties of wheat-based biscuits. An integrated approach was applied to characterize carob flour composition, including its nutritional profile, phenolic compounds, antioxidant potential, and volatile constituents, and to investigate how these characteristics influence the quality attributes of fortified biscuits.

## 2. Materials and Methods

### 2.1. Preparation of C. siliqua Pod Flour

Mature *C. siliqua* L. (carob) pods used in this study were manually harvested in August 2024 from a forest located in Eastern Algeria (Oum El Bouaghi Province, northeastern Algeria; 35.87696° N, 7.13227° E), at an altitude of approximately 980 m above sea level. The region is characterized by a semi-arid continental climate, typical of the Algerian High Plateaus, with cold winters and hot, dry summers. After harvesting, the pods were dried by air-drying at ambient conditions (25 ± 2 °C) for approximately 7 days and subsequently kibbled manually to separate the seeds from the pulp portion. The pulp was dehulled for carob powder production, ground to different particle sizes, and roasted in an electric oven (Memmert GmbH + Co. KG, Schwabach, Germany) at 130 °C for 20 min [11]. The roasted material was then milled using a mechanical grinder (Moulinex, Groupe SEB, Écully, France) to obtain a fine powder, called carob flour. The resulting flour was sieved through a test sieve with a mesh size range of 150–400 μm to ensure uniform particle size distribution and stored in glass containers at room temperature (25 ± 2 °C) in the dark until use.

### 2.2. Preparation of Biscuits

The biscuits were prepared by partially substituting refined soft wheat flour (*Triticum aestivum* L.) with wholemeal *C. siliqua* flour, prepared as above described. The soft wheat flour used in this study was a commercial product (Kenza, Constantine, Algeria) with the following composition: 10.5% protein, 1.2% fat, 73.3% carbohydrates, 2.8% fiber, 0.6% ash, 14.0% moisture. In addition to flour, the ingredients used for biscuit preparation included refined white sugar (Cevital, Béjaïa, Algeria), extra virgin olive oil (Baghlia, Boumerdès, Algeria), and food-grade sodium bicarbonate (Nouara, Ain Benian, Algeria), all purchased from local retailers. Extra virgin olive oil was selected as the lipid source due to its availability and traditional use in Mediterranean bakery products. The amount used was kept constant among formulations to evaluate the effect of carob flour incorporation while minimizing variations related to the lipid fraction.

Accordingly, three biscuit formulations were produced: a control (CTR) containing 100 g wheat flour and two formulations in which wheat flour was replaced with wholemeal *C. siliqua* flour at substitution levels of 30% (C30: 70 g wheat flour + 30 g carob flour) and 60% (C60: 40 g wheat flour + 60 g carob flour). Preliminary trials were conducted to empirically determine the amount of water required to achieve a similar dough consistency for all three biscuit types. The amount of water needed was found to be comparable for both the control and carob-enriched doughs. Therefore, the same amount of water was used for all biscuit formulations (Table 1).

The preparation process consisted of (i) mixing 100 g flour (either 100 g of wheat flour for the control biscuit or a blend of wheat flour and 30 g or 60 g/100 g of carob flour for C30 and C60 biscuits), 35 g of sugar, 14 mL of extra virgin olive oil, and 0.5 g of sodium bicarbonate using a planetary mixer (Kenwood, Havant, UK) for 7 min; (ii) adding 25 mL of drinking tap water and kneading again for 10 min; and (iii) rolling out the dough to a thickness of 6 mm with a rolling pin, shaping round biscuits (4.5 cm in diameter), and baking in a preheated electric oven (Memmert GmbH + Co. KG, Schwabach, Germany) for 15 min at 160 °C. All biscuit formulations were baked in the same electric oven under identical temperature and time conditions to minimize processing variability. The biscuit recipe and baking conditions were selected based on previous studies [12,13]. Before analyses, the biscuits were finely ground using an electric grinder (Moulinex, Groupe SEB, Écully, France), except for sensory and dimensional determination. Samples were stored in sealed food-grade bags under dry conditions at room temperature (25 ± 2 °C) until analysis.

### 2.3. Basic Physicochemical Determinations

Moisture content was determined by oven drying at 105 °C, according to the AOAC official method 934.01 [14]. Ash content was measured by incinerating flour at 550 °C in a muffle furnace (B180, Nabertherm, Lilienthal, Germany), following the AACC method [15]. Protein content (N × 5.7) was analyzed using the Kjeldahl method according to the AACC approved method 46-11.02 [16], while fat was extracted and determined by Soxhlet apparatus using diethyl ether as solvent. Total dietary fiber was assessed by the enzymatic-gravimetric procedure [17], and carbohydrates were calculated by difference. Mineral content (Cu, Mn, Zn, Fe, Na, Mg, Ca, P, and K) was determined by atomic absorption spectrophotometry following the AOAC official method [18].

Colorimetric evaluations of the red index (a*), yellow index (b*) and brown index (BI, defined as 100 L*) were carried out under D65 illuminant by using a spectro-colorimeter CM-700d (Konica Minolta Sensing, Osaka, Japan), equipped with a pulsed xenon lamp and granular material attachment CR-A50 (Konica Minolta Sensing, Osaka, Japan) [13].

### 2.4. Phenolic Compounds Extraction

Phenolic compounds were extracted according to the procedure described by Pasqualone et al. [13], with slight modifications. Briefly, 5 g of sample (carob flour or powdered biscuits) was mixed with 50 mL of methanol/water solution (80:20 *v*/*v*). The mixture was subjected to ultrasound treatment (UP400St, Hielscher Ultrasonics, Teltow, Germany) for 35 min at room temperature (25 ± 2 °C). The extracts were then centrifuged at 6000× *g* for 10 min at 4 °C and filtered through a 0.45 μm membrane filter (Sigma-Aldrich Chemical Co., St. Louis, MO, USA). The obtained extracts from carob flour and biscuit samples were used for the determination of total phenolic content (TPC) and total flavonoid content (TFC). LC-MS/MS analysis was performed only on carob flour extract for the tentative identification of phenolic compounds.

### 2.5. Total Phenolic Content (TPC) Quantification

Total phenolic content (TPC) was determined by the Folin-Ciocalteau method using a calibration curve of gallic acid (R^2^ = 0.997), according to Caponio et al. [19]. Briefly, 100 µL of appropriately diluted extract was mixed with 100 µL of Folin-Ciocalteau reagent. After 4 min, 800 µL of 5% (*w*/*v*) Na_2_CO_3_ solution was added, and the mixture was incubated in a water bath at 40 °C for 20 min. After being cooled down for 15 min, the absorbance was measured at 750 nm using a UV-Vis spectrophotometer (V-730, Jasco Inc., Tokyo, Japan). The TPC was expressed as mg of gallic acid equivalents (GAE) per 100 g of dry matter (DM).

### 2.6. Total Flavonoid Content (TFC) Quantification

Total flavonoid content (TFC) was determined spectrophotometrically, as reported in Makhlouf et al. [20]. Briefly, 500 µL of extract was mixed with 2 mL of distilled water and 150 µL of NaNO_2_ solution (5% *w*/*v*). After 6 min, 150 µL of a 10% AlCl_3_ solution was added and allowed to stand further 6 min. Thereafter, NaOH solution (2 mL, 1 M) was added, and the final volume was brought to 5 mL with distilled water. After incubation at room temperature (25 ± 2 °C) for 15 min, the absorbance of the mixture was read at 510 nm. The results were expressed as mg of quercetin equivalent (QE) per 100 g of DM, by means of a quercetin standard curve (R^2^ = 0.998).

### 2.7. LC-MS Analysis

Phenolic compounds were identified using a UHPLC Ultimate 3000 RS Dionex (Thermo Fisher Scientific, Waltham, MA, USA) interfaced by H-ESI II probe with a LTQ Velos pro linear ion trap mass spectrometer (Thermo Fisher Scientific, Waltham, MA, USA). The UHPLC system was composed of a LPG-3400 RS quaternary pump, WPS-3000 TRS autosampler, TCC-3000 RS column older, and PDA 3000 RS detector (Thermo Fisher Scientific, Waltham, MA, USA). The extract was diluted with the LC mobile phase (1:1, *v*/*v*) directly into the vial for LC-MS analysis. The sample injection volume was 5 µL.

The analytical separation of phenols was achieved using a Hypersil GOLD aQ C18 column (Thermo Fisher Scientific, Waltham, MA, USA), 100 mm of length, 2.1 mm internal diameter and 1.9 µm of particle size, held at 30 °C and at a constant flow of 0.3 mL/min. A binary gradient was obtained using 10% of formic acid in water (A) and 0.1% formic acid in acetonitrile (B), from 2% to 70% of B linear gradient in 20 min. The absorbance of eluting compounds was recorded from 220 to 600 nm. The full chromatograms at 280 and 320 nm are shown in Supplementary Figure S1. The MS conditions were capillary temperature 320 °C; source heater temperature 280 °C; nebulizer gas N_2_; sheath gas flow 30 psi; auxiliary gas flow 7 arbitrary units; capillary voltage −2800 V, S-Lens RF Level 60%. Data were acquired in negative and positive ionization modes. A data-dependent experiment was used to collect MS^2^ data. The data-dependent settings were: full scan from 200 to 1000 *m*/*z*, activation level 500 counts, isolation width 2 Da, and CID energy 35. Phenolic compounds were tentatively identified based on their molecular ions ([M + H]^+^ or [M − H]^−^), MS/MS fragmentation patterns, UV–visible absorption maxima (λ_max_), and comparison with previously reported LC-MS/MS data.

### 2.8. Determination of Antioxidant Activity

The antioxidant activity of the extracts was evaluated using two complementary assays: the ABTS (2,2′-azinobis(3-ethylbenzothiazoline-6-sulphonic acid) diammonium salt) assay and the DPPH (1,1-diphenyl-2-picrylhydrazyl) assay, as described in Pasqualone et al. [21].

The scavenging activity of the ABTS radical was assessed by mixing 50 µL of each extract with 950 µL of the ABTS reagent previously prepared. After 8 min, the decrease in absorbance was measured at 734 nm. A calibration curve of 6-hydroxy-2,5,7,8-tetramethylchroman-2-carboxylic acid (Trolox) was used to quantify the antioxidant activity, and the results were expressed as µmol of Trolox equivalents (TE) per g of dry matter (µmol TE/g DM).

For DPPH radical scavenging activity, 50 µL of each extract was added to 950 µL of a DPPH solution (0.08 mM in ethanol). After incubation in the dark for 30 min, the absorbance was measured at 517 nm. The results were expressed as μmol TE per g of dry matter (µmol TE/g DM).

### 2.9. Determination of Induction Time

The oxidative stability of carob flour, specifically the stability of its lipid fraction, was assessed by measuring the induction time (IT) using an automated testing device (Rapidoxy, Anton Paar, Blankenfelde-Mahlow, Germany). The instrument induces a forced oxidation by subjecting the sample (3 g) to conditions of high temperature and high oxygen pressure [13]. IT is defined as the time required for a 10% drop in oxygen pressure under the following conditions: temperature = 140 °C, initial oxygen pressure = 700 kPa.

### 2.10. Determination of Volatile Compounds by GC-MS

Volatile compounds in carob flour were determined by head space solid-phase micro extraction (HS-SPME) coupled to gas chromatography–mass spectrometry (GC-MS), using a head space temperature of 45 °C and a contact time of 40 min. 710.7 mg of powdered dry sample was directly weighed into the headspace vial, and chromatographic separation was performed on a polar wax capillary column (SC-WAX; 30 m × 0.25 mm i.d., 0.25 µm film thickness; maximum operating temperature 250 °C). The injector temperature was set at 270 °C. The GC oven temperature program was as follows: initial temperature 30 °C (held for 1 min), then increased at 10 °C/min to 200 °C and held for 1 min (total run time 19 min).

Compound identification was based on comparison of acquired mass spectra with those in a mass spectral library and corroborated by retention information using Kovats retention indices (RI). Reference RI values were taken from the NIST “Gas Chromatographic Retention Data” database (National Institute of Standards and Technology, U.S. Department of Commerce; available at: https://webbook.nist.gov/chemistry/gc-ri/; accessed on 25 February 2026). For RI determination, a commercial standard mixture of saturated n-alkanes (C7–C40) (provided by Merck; Darmstadt, Germany), analyzed under the same chromatographic conditions, was used as the external reference series. The volatile compounds were expressed as relative peak area percentages (%), representing the relative contribution of each compound to the total detected volatile fraction.

### 2.11. Determination of Dimensional Indices of Biscuits

Diameter (D) and thickness (T) of biscuits were measured both before and after baking using a caliper. These values were used to calculate the surface area (S) and volume (V). The percentage increase in D, T, S, and V due to baking was calculated using the following formula:

$$\%\;of\;increase\;in\;D\;\left(or\;T,S,V\right)=\frac{D\;\left(or\;T,S,V\right)\;after\;baking-D\;\left(or\;T,S,V\right)\;before\;baking}{D\;\left(or\;T,S,V\right)\;before\;baking}\times 100$$

Spread factor was calculated as the ratio of the diameter to the thickness of baked biscuits, according to the AACC Method 10–50.05 [22]. Six biscuits were analyzed for each parameter.

### 2.12. Sensory Evaluation of Biscuits

A Quantitative Descriptive Sensory Analysis (QDA) was conducted following the procedure described in previous studies [13,23]. The evaluation was performed using a trained panel of eight members (four males and four females, aged between 23 and 45 years), previously selected based on their reliability, consistency, and discriminative ability. All participants were healthy adults without reported food allergies or sensory impairments affecting taste or smell. The selected panelists were regular consumers of bakery products, including biscuits. Before the formal evaluation, training sessions were conducted using biscuit samples to familiarize the panelists with the sensory descriptors, their definitions, and the use of the intensity scale.

The sensory descriptors were selected based on previous studies on bakery products and refined during the training sessions by consensus among the panelists to ensure that each descriptor was clearly defined, relevant to carob-enriched biscuits, and consistently interpreted by all assessors. Following the principles of QDA, each sensory attribute was evaluated independently to establish a quantitative sensory profile of the different biscuit formulations. The evaluated attributes included visual-tactile characteristics (color and friability), odor attributes (bran-like odor and caramel odor), taste attributes (sweetness and bitterness), and mouthfeel-related texture attributes (dryness, astringency, and graininess). Each descriptor was rated on an anchored line scale from 0 to 9 (0 = minimum intensity, 9 = maximum intensity). Biscuit samples from each formulation were randomly coded using three-digit numbers and presented to panelists in a randomized serving order to minimize positional effects. The evaluations were carried out in a quiet sensory evaluation room under controlled environmental conditions, with each panelist assessing the samples individually to minimize distractions and avoid interactions among assessors. Water was provided for palate cleansing between samples. A total of three sensory sessions were conducted, and the same trained panel was used throughout all sensory sessions to ensure consistency in attribute evaluation. All participants provided informed consent before participation. The sensory evaluation was conducted in accordance with the institutional ethical requirements, and panelists’ confidentiality was ensured.

### 2.13. Statistical Analysis

For chemical analyses, one biscuit batch was prepared for each formulation (CTR, C30, and C60), and analytical determinations were performed in triplicate. For dimensional measurements and sensory evaluation, three independent biscuit preparations were conducted for each formulation. Data were expressed as mean ± standard deviation (SD). Statistical analysis was carried out using XLSTAT software 2019 (Addinsoft SARL, New York, NY, USA). For sensory evaluation, the intensity scores obtained for each QDA descriptor were analyzed individually. Significant differences were determined at *p* ≤ 0.05 by one-way analysis of variance (ANOVA), followed by Tukey’s HSD test.

## 3. Results and Discussion

### 3.1. Physicochemical and Nutritional Characteristics of C. siliqua Flour

The chemical composition of the *C. siliqua* flour used in the biscuit formulations is presented in Table 2. The moisture content of the flour was relatively low, which is suitable for ensuring a good shelf life and product stability.

The ash content of carob flour was found to be 2.40 g/100 g, slightly higher than the typical range reported by Menard et al. [24] for biscuit-making flours. The carob flour used in this study had a relatively low protein content, which remained within acceptable limits for biscuit production. In wheat-based formulations, protein levels above 12 g/100 g favor excessive gluten network development, negatively affecting biscuit texture [24,25].

However, carob flour does not contain gluten-forming proteins; therefore, its incorporation results in gluten dilution, reducing dough strength and improving biscuit friability. The fat content of carob flour was notably low, aligning with the naturally low lipid profile of carob pods.

Carob flour also demonstrated a very high total dietary fiber content, significantly exceeding the fiber levels found in conventional flours, which typically range between 2 and 12 g/100 g [2]. The high fiber content not only offers nutritional benefits but also influences texture, moisture retention, and the structural characteristics of baked goods, making carob flour particularly well-suited for high-fiber and functional food formulations [26].

Carbohydrates made up the main fraction of carob flour, which is expected given the high levels of natural sugars and non-starch polysaccharides in carob [27]. This carbohydrate content contributes to the flour’s inherent sweetness and energy value, which can enhance the flavor of baked goods without added sugars.

Overall, the nutritional composition of carob flour in this study is generally consistent with values reported in the literature. Protein and ash contents align with findings by Amer et al. [28] and Benković et al. [29], while the fat content remains within the low range noted in previous studies. The total dietary fiber content is notably higher compared to literature findings, particularly those of Hafez et al. [30]. These variations may be due to genetic, environmental, and climatic factors, as well as differences in harvesting time [31].

In terms of mineral content, carob flour proved to be a rich source of essential minerals, particularly potassium (927 mg/100 g) and calcium (319 mg/100 g), along with magnesium, phosphorus, and iron. These minerals are critical for various enzymatic processes and antioxidant functions [27]. The results are consistent with previous studies reported by Özcan et al. [32] and Oziyci et al. [33]. Hence, the addition of carob flour to wheat flour in food preparations might result in a mineral fortification, which depends on the amount added, but that can have interesting implications from the nutritional point of view. Such incorporation may enhance the mineral profile of cereal-based products, which could be nutritionally relevant for consumers with increased mineral requirements.

The color analysis of *C. siliqua* flour revealed moderate red (a*) and high yellow (b*) indices, with a high brown index (61.05), indicating the naturally dark tone of carob flour. This color profile, mainly associated with natural pigments, phenolic compounds, and roasting-derived Maillard products, affects the appearance of final baked products and is characteristic of carob-based goods [12]. Such color characteristics can be advantageous in wholegrain-style formulations, where a darker tone is often associated with fiber richness and higher nutritional value [34].

The roasting process applied during carob flour preparation may have influenced some of the physicochemical characteristics observed in the present study. Thermal treatment promotes moisture reduction, which may contribute to improved flour stability during storage, and induces non-enzymatic browning reactions, including Maillard reactions and caramelization, responsible for the characteristic dark color of roasted carob flour [2,8,11,35]. Moreover, roasting can induce structural modifications of carbohydrates and other macromolecules, potentially affecting the functional properties and technological behavior of the flour [2,35]. Therefore, differences between the composition values reported in this study and those described for raw carob flour may be related not only to cultivar, geographical origin, and harvesting conditions but also to processing parameters, particularly roasting intensity.

### 3.2. Phenolic Compounds, Antioxidant Properties and Phenolic Profile of C. siliqua Flour

Carob flour exhibited remarkably high total phenolic content (875.4 mg GAE/100 g) and total flavonoid content (230.2 mg QE/100 g), together with strong antioxidant capacity as measured by DPPH (50.8 µmol TE/g) and ABTS (72.5 µmol TE/g) assays (Table 2). These results are consistent with previous reports highlighting *C. siliqua* as a valuable source of bioactive compounds with antioxidant properties [36,37]. The relatively high phenolic and flavonoid contents observed in the present study may also be influenced by the roasting conditions. Moderate thermal treatment has been reported to enhance the recovery of phenolic compounds, while excessive roasting may induce the degradation of thermolabile phenolics, particularly flavonoid glycosides [11,35]. Thus, the selected roasting conditions likely contributed to preserving a high proportion of phenolic constituents in the roasted carob flour. Accordingly, an induction time of 117.84 min was observed, which suggests a moderate level of oxidative stability and, in turn, is also likely linked with the low-fat content observed that reduces the likelihood of rancidity during storage [38].

The *C. siliqua* flour extract was also qualitatively analyzed by LC-MS/MS to tentatively identify its phenolic constituents. Table 3 summarizes the peaks detected, along with their retention times (RT), λ_max_, mass spectral data ([M + H]^+^, [M − H]^−^, MS/MS fragments), tentative identifications and supporting literature references. A total of 21 compounds were detected, of which 14 were tentatively identified based on comparison with previously reported data. The identified compounds belonged to various phenolic classes, predominantly flavonoids and phenolic acid derivatives, as well as carbohydrate derivatives and hydrolysable tannins.

Among the identified compounds, flavonoids represented the largest group of identified phenolics, with several derivatives of quercetin, myricetin, and naringenin detected. For instance, peak 4 was tentatively identified as methylapigenin-O-pentoside, flavonoid glycoside previously reported as characteristic constituents of *C. siliqua* pods [39]. Peak 5, *m*/*z* 511.14, was assigned as an acylated dihexose flavonoid, consistent with the structures reported by Ghorbaninejad et al. [40]. Additional flavonoids included naringenin-O-hexoside (peak 8), and several flavonol glycosides (peaks 9–11, 15, and 17), primarily derived from quercetin and myricetin. These compounds are well-documented in *C. siliqua* pods [41,42]. Notably, peak 18 (*m*/*z* 472.39) was identified as quercetin gallate, a galloylated flavonol previously reported in carob extracts [43].

Phenolic acids were also well-represented. Peak 7 (*m*/*z* 393.26) was tentatively assigned as a caffeic acid derivative, while peak 20 (*m*/*z* 350.20) was identified as gallic acid glucoside, a hydroxybenzoic acid derivative commonly found in carob tissues [41]. Regarding hydrolyzable tannins, peak 12 (*m*/*z* 481.32) was identified as digalloyl hexose, consistent with gallotannins previously described in *C. siliqua* [39]. Several peaks were attributed to carbohydrate derivatives, including allyl disaccharide (Peak 1, *m*/*z* 381.07), (iso)butyryl hexose-pentose (Peak 6, *m*/*z* 425.09), and acylated dihexose (Peak 5, *m*/*z* 511.14).

Seven peaks (2, 3, 13, 14, 16, 19, 21) could not be matched to existing literature data for which further structural elucidation is required.

Overall, LC-MS/MS profile confirmed the presence of a broad spectrum of phenolic compounds in carob flour. The identified phenolic classes, particularly flavonoid glycosides, phenolic acids, and tannin derivatives, provide a plausible explanation for the antioxidant potential observed in the present study. These bioactive constituents are therefore likely to contribute to the antioxidant capacity of carob flour and support its value as a functional ingredient. Moreover, the detection of hydrolyzable tannins and sugar-phenolic conjugates, together with roasting-induced chemical transformations, may also influence the sensory profile, particularly astringency and mouthfeel. These findings align with previous studies [39,41,43], highlighting *C. siliqua* flour as a promising source of health-promoting compounds for nutraceutical and functional food applications.

**Table 3:** Tentative identification of phenolic compounds in *C. siliqua* flour by LC-MS/MS.

Peak No.	λ_max_ (nm)	RT (min)	Identified Compounds *	Class	[M+H]^+^ or [M-H]^−^ (*m*/*z*)	MS/MS (*m*/*z*)	Reference
1	280	0.89	Allyl disaccharide	Carbohydrate derivative	381.07	218.97; 200.97	[43]
2	280	1.11	Unknown	–	365.11	202.99	–
3	280	1.18	Unknown	–	365.13	202.99; 184.99	–
4	280	5.13	Methylapigenin-O-pentoside	Flavonoid	419.21	317.01	[39]
5	290	5.63	Acylated dihexose	Carbohydrate derivative	511.14	317.03	[40]
6	280	6.40	(Iso)Butyryl hexose-pentose	Carbohydrate derivative	425.09	255.02	[44]
7	270	6.82	Caffeic acid derivative	Phenolic acid derivative	393.26	393.19; 302.90	[45]
8	280	7.18	Naringenin-O-hexoside	Flavanone	437.26	437.18; 368.88	[39]
9	266	7.33	Quercetin pentoside	Flavonol	433.15	317.02	[44]
10	254	7.54	Quercetin-O-hexoside	Flavonol	463.18	301.07	[42]
11	284	7.64	Myricetin-3-O-α-L-rhamnopyranoside	Flavonol	465.09	317.02	[41]
12	260	7.83	Digalloyl hexose	Hydrolyzable tannin	481.32	481.22; 437.19	[39]
13	280	8.08	Unknown	–	467.23	449.16	–
14	280	8.30	Unknown	–	525.24	525.20	–
15	260	8.54	Myricetin-O-hexoside	Flavonol	475.13	387.09	[39]
16	270	8.78	Unknown	–	603.19	441.06	–
17	280	10.50	Myricetin pentoside	Flavonol	450.39	433.12	[41]
18	260	10.68	Quercetin gallate	Flavonol	472.39	404.97; 455.22	[43]
19	260	10.95	Unknown	–	207.22	151.06	–
20	260	12.62	Gallic acid glucoside	Phenolic acid derivative	350.20	332.17	[41]
21	260	21.65	Unknown	–	795.36	675.40	–

RT: Retention time; MS: Mass spectrometry; * tentatively identified using mass spectra and λ_max_ with comparison to previously reported LC-MS/MS data.

### 3.3. Volatile Compounds of C. siliqua Flour

**Figure 1:**
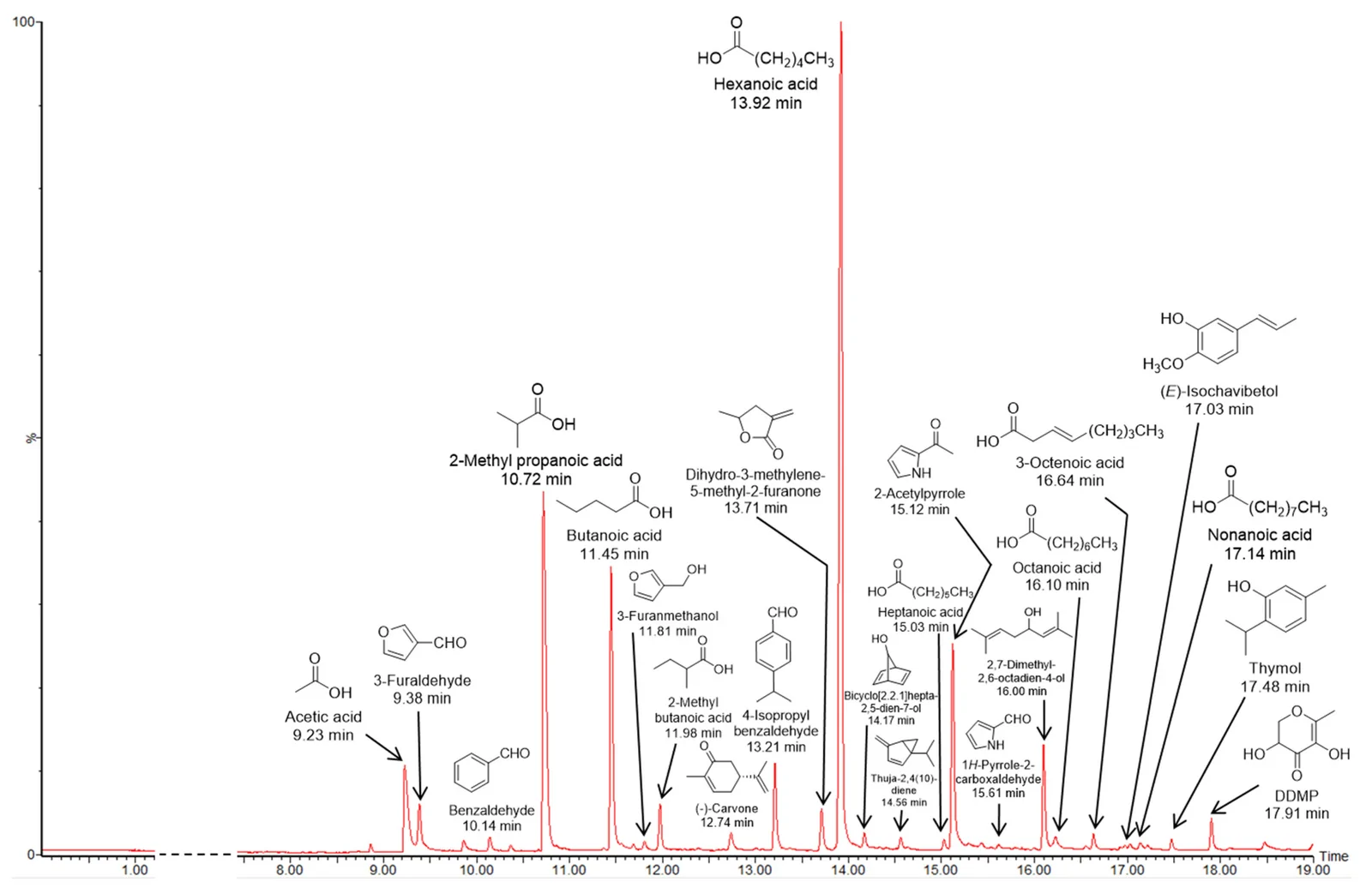
Chromatogram obtained from the GC–MS analysis of *C. siliqua* and chemical structures and retetion times of the volatile compounds identified.

The volatile compounds in *C. siliqua* flour were extracted by SPME and analyzed by GC-MS, yielding a well-resolved chromatographic profile, as shown in Figure 1.

Table 4 summarizes the identified compounds, along with their experimental Kovats indices, retention times, relative areas, molecular formulas, and chemical classes. A total of twenty-four compounds were identified. Results revealed a diverse profile of volatile compounds belonging to several chemical classes, namely acids, aldehydes, ketones, alcohols, terpenoids, furans, and phenolics.

Among these, acids dominated, with hexanoic acid (37.50%), 2-methyl propanoic acid (18.20%), and butanoic acid (10.90%) being the most abundant. Aldehydes, ketones, alcohols, terpenoids, phenolics, and furans were also detected. The relative abundance of carboxylic acids, even at low concentrations, is consistent with previous reports on carob powder, where acids increase during pod ripening and become the dominant class of volatiles [46,47].

Aldehydes were also detected, albeit at lower levels, with furfural (2.90%) and benzaldehyde (0.70%) as key representatives. Their occurrence indicates thermal or oxidative degradation of sugars and amino acids through Maillard and caramelization pathways [48,49]. Similar aldehydes have been identified in both roasted and raw carob, where benzaldehyde, in particular, is recognized as a major odorant responsible for its cocoa-like perception [44].

Ketones, primarily 2-acetylpyrrole (8.80%), and alcohols such as furfuryl alcohol (0.40%), also point to Maillard-related transformations. Although quantitatively minor, these compounds are sensorially potent and play an important role in flavor due to their low odor thresholds reported for furans, aldehydes, and Maillard-derived compounds.

Terpenoids and phenolics, though only detected in trace amounts (≤1.00%), such as carvone, thymol, and isochavibetol, are notable due to their strong odor thresholds and their known role in conferring herbal, spicy, and medicinal nuances [48,50]. Finally, furanic compounds, including 2,3-dihydro-3,5-dihydroxy-6-methyl-4H-pyran-4-one (DDMP), were also detected at low concentrations.

These compounds are typically generated via Maillard reactions and are associated with sweet, caramel-like, and fruity notes. Their presence aligns with previous findings on roasted carob, where furans are considered as key contributors to its characteristic chocolate-like aroma [48]. In accordance with these observations, *C. siliqua* flour presented a sweet, mild flavor with subtle nutty and caramel notes as empirically evaluated by tasting. Its flavor resembled that of chocolate, offering a smooth and pleasant overall taste without pronounced bitterness typically associated with cocoa-based products.

The chemical profile observed by GC–MS provides insight into the sensory attributes of carob flour. Organic acids represented a major proportion of the detected volatile fraction based on relative peak area values, attending at values gathered in Table 4 (these values reflect the relative contribution of each compound within the volatile profile and must not be interpreted in absolute concentration terms). In sensory terms, organic acids may contribute to acidic, fatty, or cheesy notes, which in small amounts can add complexity and balance the natural sweetness of biscuits but may cause off-flavors at higher concentrations [47]. Conversely, Maillard-derived aldehydes (furfural, benzaldehyde, 4-isopropylbenzaldehyde) and ketones (2-acetylpyrrole) are especially relevant to sensory perception. These compounds are associated with roasted, caramel, nutty, and cocoa-like aromas typically desirable in baked goods [51]. In particular, 2-acetylpyrrole and furfuryl alcohol may contribute to the roasted and chocolate-like notes of carob flour, underscoring its potential role as a natural cocoa substitute [48,50]. The contribution of terpenoids and phenolics, even in trace amounts, should not be underestimated, as compounds such as carvone and thymol provide minty, herbal, and clove-like nuances, adding further depth to the aroma profile [50]. Meanwhile, furans like DDMP bring malt-like, caramel, and roasted notes, which are often critical for consumer acceptance of roasted carob flour [4,47,52]. Overall, this combination may explain the distinctive cocoa-like, roasted, and slightly acidic sensory flavor of carob flour and supports its potential as a natural flavor-enhancing ingredient in bakery products.

### 3.4. Effect of Carob Flour Incorporation on Phenolic Content and Antioxidant Properties of Biscuits

Table 5 reports the antioxidants content and activity of the biscuits. As expected, the control biscuit showed the lowest values of TPC, TFC, DPPH, and ABTS, reflecting the absence of carob flour in its formulation. On the other hand, incorporation of carob flour significantly enhanced the antioxidant profile of all biscuit formulations in an, almost linear, dose-dependent manner. The C60 biscuit, containing 60% carob flour, exhibited the highest antioxidant parameters among all biscuit samples, followed by C30. The reduction in antioxidant content compared to the flour may be attributed to oxidative reactions during dough preparation and thermal degradation during baking [53]. Nevertheless, carob-containing biscuits retained appreciable levels of antioxidant compounds, as evidenced by their significantly higher DPPH and ABTS values compared to the control biscuit. These findings demonstrate that carob flour not only improves the nutritional quality of biscuits by increasing phenolic content, but also might contribute to enhancing oxidative stability, and shelf-life, in the final product [36,54].

### 3.5. Dimensional Characteristics of Biscuits

Several dimensional indices were measured in the formulated biscuits to evaluate the extent of thermal expansion during baking which, in turn, can be associated with the technological properties of the dough [13]. The results in Table 6 demonstrate that substituting wheat flour with carob flour significantly influenced the dimensional properties of the biscuits after baking.

The diameter of the biscuits increased significantly with the level of carob flour substitution, with biscuits appearing progressively larger as the carob flour content increased from 0% (Control) to 60% (C60). This effect may be linked to the reduced dough elasticity and gas retention capacity associated with lower gluten content [13]. The progressive replacement of wheat flour by gluten-free carob flour diluted the gluten-forming proteins, resulting in a weaker and less cohesive dough structure. Such gluten weakening reduces dough resistance during baking, thereby facilitating lateral expansion of the biscuits [55].

The increase in thickness was less pronounced and showed only slight, though statistically significant, changes between formulations. This modest increase may reflect the denser structure of carob flour compared to wheat flour, which might limit vertical rise but still contribute to overall expansion. Moreover, the high dietary fiber content of carob flour can compete with starch and gluten proteins for water, modifying dough hydration and limiting the development of a continuous gluten network. Consequently, dough expansion tends to occur preferentially in the horizontal rather than the vertical direction [55,56].

The volume increase followed a similar trend, with C60 biscuits exhibiting the highest values. However, volume expansion was less influenced by the level of substitution, possibly due to the limited effect on thickness. The spread factor, a key quality parameter in biscuits, also increased significantly with carob flour addition.

A higher spread factor generally correlates with improved biscuit quality and consumer acceptability [57]. The control biscuit, made solely with wheat flour, had the lowest spread factor, while the highest was recorded in the C60 formulation. This increase is likely due to the reduced gluten structure, which facilitates horizontal expansion during baking. It is well established that strong gluten networks can restrict biscuit spread and result in thicker, less uniform products [58].

In addition to gluten dilution, partial replacement of wheat flour by carob flour also reduced the relative starch content of the formulations. This starch dilution, together with the high water-binding capacity of dietary fiber, modifies dough rheology by decreasing dough cohesiveness and increasing its tendency to spread during baking [56]. Furthermore, soluble fibers and polyphenols present in carob flour may interact with gluten proteins and starch granules, altering the viscoelastic behavior of the dough and contributing to the observed dimensional changes. These combined mechanisms explain the progressive increase in biscuit diameter, surface area, and spread factor as the substitution level increased.

### 3.6. Sensory Evaluation of Carob-Enriched Biscuits

The sensory analysis of biscuits formulated with increasing levels of *C. siliqua* flour (C30 and C60) revealed significant differences (*p* ≤ 0.05) in most evaluated sensory parameters compared to the control (Table 6). The incorporation of carob flour notably influenced the color, odor, flavor, and overall sensory acceptability of the products.

Color intensity increased progressively with carob addition, resulting in a darker appearance. This change reflects the natural pigments of carob flour as well as the formation of Maillard-derived melanoidins during baking. Conversely, friability decreased significantly with increasing carob substitution, indicating a denser and less crumbly texture in high-carob formulations. This could be due to the higher fiber and sugar content of carob flour, which tends to reduce gluten network formation and increase dough viscosity [59].

Despite the reduced friability, the biscuits remained acceptable in texture. In terms of aroma and flavor, carob incorporation significantly enhanced bran-like, caramel, nutty, and chocolate-like notes. These sensory improvements are consistent with the GC–MS results, which revealed the presence of Maillard-derived volatiles such as furfural, furfuryl alcohol, and 2-acetylpyrrole, compounds associated with roasted and cocoa-like aromas [47]. Additionally, furans like DDMP contributed sweet, malt-like, and caramel nuances, enhancing the perceived sweetness [60].

The increase in sweet taste in C30 and C60 formulations can also be attributed to the natural sugars in carob pulp (sucrose and fructose), which impart pleasant sweetness while reducing the need for added sugars [5,48]. Interestingly, no bitterness or astringency was detected in any sample, despite the high total phenolic content, indicating that carob flour addition did not introduce undesirable off-flavors. This effect is likely related to the qualitative profile of carob phenolic compounds, as well as to the roasting process.

A slight increase in dryness was observed with higher carob content, which can be linked to the fiber’s moisture-binding capacity, though this did not negatively affect overall perception.

Finally, the C30 formulation had the highest overall acceptability score, suggesting that moderate substitution (30%) provided the best balance between flavor, texture, and aroma, even more than in the control biscuits. At higher substitution levels (C60), although the darker color, enhanced sweetness, and more intense roasted, caramel, and cocoa-like aromas contributed positively to sensory perception, these improvements were not sufficient to compensate for the loss of friability and the slight increase in dryness. In biscuit products, texture-related attributes such as friability and mouthfeel play a critical role in consumer acceptance; therefore, the denser texture of C60 likely reduced eating pleasure despite its richer aroma profile. Consequently, C30 appears to represent an optimal compromise between the desirable sensory contributions of carob flour and the preservation of preferred biscuit texture. These observations agree with previous studies showing that partial replacement of wheat flour with carob (20–40%) enhances sensory appeal, whereas excessive substitution may affect texture [2,61].

## 4. Limitations and Future Perspectives

Although this study provides a comprehensive evaluation of roasted carob flour and its application in wheat-based biscuits, some limitations should be acknowledged. The analytical strategy was designed to characterize the intrinsic nutritional and physicochemical properties of carob flour as the functional ingredient, while the effects of its incorporation were assessed in biscuits through technological, sensory, phenolic, antioxidant, and volatile analyses. Consequently, proximate composition and mineral analyses were not repeated in the biscuit formulations, as the primary objective was to evaluate the impact of carob flour incorporation rather than to comprehensively characterize both matrices. Future studies should investigate nutrient and bioactive compound retention during processing, as well as dough rheology, shelf-life stability, consumer acceptance using larger panels, and the feasibility of industrial-scale production. Furthermore, during this work the same trained sensory panel was used throughout all evaluation sessions. Although this approach is commonly adopted in QDA studies to improve consistency and reduce inter-panel variability, future studies could include additional trained panels or larger sensory panels to further assess the reproducibility and robustness of the sensory results.

## 5. Conclusions

The integration of *C. siliqua* flour into biscuit formulations significantly enhanced their nutritional, functional, and sensory qualities. The characterization of carob flour revealed its high dietary fiber content, valuable mineral composition, and richness in phenolic compounds associated with antioxidant potential. The combined GC–MS and LC–MS analyses further highlighted the contribution of Maillard-derived volatiles and phenolic compounds to the distinctive roasted, caramel, and cocoa-like characteristics of carob flour. The 30% substitution level provided the most balanced product in terms of texture, sweetness, flavor, and overall acceptability. These findings highlight the potential of roasted carob flour as a promising ingredient for nutritionally enriched bakery products, support its use as a natural cocoa alternative, and contribute to the valorization of this underutilized Mediterranean resource. Nevertheless, further studies are needed to evaluate shelf life, dough rheology, consumer acceptance, and the feasibility of industrial-scale production.

## Figures and Tables

**Table 1:** Formulation of control and carob-enriched biscuits.

Ingredients	CTR	C30	C60
Wheat flour (g)	100	70	40
Carob flour (g)	0	30	60
Sucrose (g)	35	35	35
Extra virgin olive oil (mL)	14	14	14
Sodium bicarbonate (g)	0.5	0.5	0.5
Drinking tap water (mL)	25	25	25

All formulations were prepared on a 100 g flour basis.

**Table 2:** Chemical composition, colorimetric properties, and antioxidant characteristics of *C. siliqua* flour.

Proximate Composition (g/100 g FW)	
Moisture content	6.20 ± 0.43
Ash content	2.40 ± 0.05
Protein content	4.20 ± 0.81
Fat	0.68 ± 0.01
Total dietary fiber	39.60 ± 0.78
Carbohydrates	46.92 ± 2.33
**Mineral content (mg/100 g FW)**	
Calcium (Ca)	319.10 ± 3.44
Potassium (K)	927.23 ± 4.56
Magnesium (Mg)	55.30 ± 2.49
Sodium (Na)	22.52 ± 2.12
Phosphorus (P)	60.40 ± 3.74
Iron (Fe)	3.40 ± 0.23
Zinc (Zn)	0.70 ± 0.00
Copper (Cu)	0.40 ± 0.00
Manganese (Mn)	0.80 ± 0.00
**Colorimetric data**	
Red index (a*)	5.81 ± 0.14
Yellow index (b*)	15.28 ± 0.32
Brown index (100 − L*)	61.05 ± 0.62
**Antioxidant and stability data**	
TPC (mg GAE/100 g)	875.40 ± 12.30
TFC (mg QE/100 g)	230.20 ± 8.10
DPPH (µmol TE/g)	50.80 ± 1.20
ABTS (µmol TE/g)	72.50 ± 1.50
Induction time (min)	117.84 ± 1.00

Values are expressed as mean of three replications ± SD.

**Table 4:** Volatile compounds identified in *C. siliqua* flour by GC–MS.

Class	Compound Annotations	Kovats Index	Retention Time (min)	Relative Area (%)	Molecular Formula
**Organic acids**	Acetic acid	1423	9.23	5.40	C_2_H_4_O_2_
	2-Methylpropanoic acid	1541	10.72	18.20	C_4_H_8_O_2_
	Butanoic acid	1601	11.45	10.90	C_4_H_8_O_2_
	2-Methylbutanoic acid	1646	11.98	1.60	C_5_H_10_O_2_
	Hexanoic acid	1819	13.92	37.50	C_6_H_12_O_2_
	Heptanoic acid	1925	15.03	0.30	C_7_H_14_O_2_
	Octanoic acid	2032	16.10	3.60	C_8_H_16_O_2_
	3-Octenoic acid	2088	16.64	0.50	C_8_H_14_O_2_
	Nonanoic acid	2140	17.14	0.30	C_9_H_18_O_2_
**Aldehydes**	3-Furaldehyde (Furfural)	1435	9.38	2.90	C_5_H_4_O_2_
	Benzaldehyde	1494	10.14	0.70	C_7_H_6_O
	4-Isopropylbenzaldehyde	1754	13.21	3.10	C_10_H_12_O
	1H-Pyrrole-2-carboxaldehyde	1982	15.61	0.20	C_5_H_5_NO
**Ketones**	2-(1-Methyl-3-butenyl)cyclohexanone	1736	13.01	0.10	C_11_H_18_O
	2-Acetylpyrrole	1934	15.12	8.80	C_6_H_7_NO
**Alcohols**	3-Furanmethanol	1632	11.81	0.40	C_5_H_6_O_2_
	2,7-Dimethyl-2,6-octadien-4-ol	2022	16.00	0.10	C_10_H_16_O
**Terpenoids**	(–)-Carvone	1712	12.74	0.90	C_10_H_14_O
	Thuja-2,4(10)-diene	1879	14.56	0.40	C_10_H_16_
	Thymol	2177	17.48	0.40	C_10_H_14_O
**Furans**	Dihydro-3-methylene-5-methyl-2-furanone	1799	13.71	1.80	C_6_H_6_O_2_
	2,3-Dihydro-3,5-dihydroxy-6-methyl-4H-pyran-4-one (DDMP)	2222	17.91	1.10	C_6_H_8_O_4_
**Phenolics**	(E)-Isochavibetol	2129	17.03	0.20	C_10_H_12_O_2_
**Others**	Bicyclo[2.2.1]hepta-2,5-dien-7-ol	1842	14.17	0.50	C_7_H_8_O

**Table 5:** Total phenolic content, total flavonoid content, and antioxidant capacity of *C. siliqua* biscuit samples.

Sample	TPC (mg GAE/100 g)	TFC (mg QE/100 g)	DPPH (µmol TE/g)	ABTS (µmol TE/g)
**Control**	187.3 ± 5.6 ^c^	21.6 ± 3.4 ^c^	10.2 ± 0.9 ^c^	13.7 ± 0.7 ^c^
**C30**	442.7 ± 9.8 ^b^	79.4 ± 6.2 ^b^	22.9 ± 1.1 ^b^	34.2 ± 1.0 ^b^
**C60**	575.2 ± 11.0 ^a^	98.6 ± 7.5 ^a^	34.5 ± 1.3 ^a^	52.1 ± 1.3 ^a^

TPC: total phenolic content; TFC: total flavonoid content; GAE: gallic acid equivalents; QE: quercetin equivalents; TE: Trolox equivalents; DPPH: 2,2-diphenyl-1-picrylhydrazyl radical; ABTS: 2,2′-azinobis(3-ethylbenzothiazoline-6-sulphonic acid). Values are expressed as mean ± standard deviation. Different letters in the same column indicate significant differences (*p* ≤ 0.05) according to one-way ANOVA followed by Tukey’s HSD test.

**Table 6:** Dimensional parameters and sensory profile of formulated biscuits.

Parameter	Control	C30	C60
**Dimensional parameters after baking**			
**Diameter increase (%)**	10.5 ± 1.2 ^c^	12.3 ± 1.4 ^b^	14.0 ± 1.6 ^a^
**Thickness increase (%)**	8.2 ± 1.0 ^b^	9.0 ± 1.1 ^ab^	9.7 ± 1.2 ^a^
**Surface increase (%)**	15.0 ± 1.8 ^c^	17.5 ± 2.0 ^b^	20.0 ± 2.2 ^a^
**Volume increase (%)**	18.0 ± 2.0 ^c^	21.0 ± 2.3 ^b^	24.0 ± 2.5 ^a^
**Spread factor**	1.50 ± 0.1 ^c^	1.60 ± 0.1 ^b^	1.70 ± 0.1 ^a^
**Sensory characteristic**			
**Color**	3.0 ± 0.4 ^c^	7.8 ± 0.3 ^b^	8.5 ± 0.2 ^a^
**Friability**	7.2 ± 0.3 ^a^	6.0 ± 0.3 ^b^	4.5 ± 0.3 ^c^
**Bran-like Odor**	1.5 ± 0.2 ^c^	4.0 ± 0.2 ^b^	6.0 ± 0.2 ^a^
**Caramel Odor**	2.5 ± 0.3 ^c^	5.7 ± 0.2 ^b^	6.5 ± 0.3 ^a^
**Sweet Taste**	5.5 ± 0.2 ^c^	7.3 ± 0.3 ^b^	8.5 ± 0.3 ^a^
**Bitter Taste**	0.0 ± 0.0	0.0 ± 0.0	0.0 ± 0.0
**Dryness**	3.0 ± 0.2 ^c^	3.5 ± 0.2 ^b^	4.0 ± 0.3 ^a^
**Astringency**	0.0 ± 0.0	0.0 ± 0.0	0.0 ± 0.0
**Chocolate-like flavor**	0.0 ± 0.0 ^c^	4.5 ± 0.2 ^b^	5.8 ± 0.3 ^a^
**Nutty**	1.0 ± 0.0 ^c^	3.5 ± 0.2 ^b^	5.0 ± 0.3 ^a^
**Overall acceptability**	4.5 ± 0.4 ^c^	8.0 ± 0.4 ^a^	6.0 ± 0.4 ^b^

Values are expressed as mean ± SD. Different letters in a row indicate significant differences (*p* ≤ 0.05) according to one-way ANOVA followed by Tukey’s HSD test.

## Data Availability

The original contributions presented in this study are included in the article/Supplementary Materials. Further inquiries can be directed to the corresponding authors.

## Supplementary Materials

The following supporting information can be downloaded at: https://www.mdpi.com/article/10.3390/foods15162785/s1, Figure S1: LC-PDA chromatograms at 280 and 320 nm of carob flour phenolic extract.

## Abbreviations

ABTS	2,2′-Azino-bis(3-ethylbenzothiazoline-6-sulfonic acid)
AACC	American Association of Cereal Chemists
AOAC	Association of Official Analytical Chemists
ANOVA	Analysis of Variance
C30	Biscuit formulation containing 30% carob flour substitution
C60	Biscuit formulation containing 60% carob flour substitution
CTR	Control biscuit without carob flour
DM	Dry Matter
DPPH	2,2-Diphenyl-1-picrylhydrazyl
DPPH	2,2-Diphenyl-1-picrylhydrazyl radical
GC-MS	Gas Chromatography–Mass Spectrometry
GAE	Gallic Acid Equivalents
HS-SPME	Headspace Solid-Phase Microextraction
IT	Induction Time
LC-MS/MS	Liquid Chromatography–Tandem Mass Spectrometry
MS	Mass Spectrometry
MS/MS	Tandem Mass Spectrometry
QDA	Quantitative Descriptive Sensory Analysis
QE	Quercetin Equivalents
RI	Retention Index
RT	Retention Time
SD	Standard Deviation
SPME	Solid-Phase Microextraction
TFC	Total Flavonoid Content
TPC	Total Phenolic Content
TE	Trolox Equivalents
