# Supplementary material for: Physicochemical and Sensory Profiling of Carob Flour and Effects of Its Incorporation on Quality Characteristics of Wheat-Based Biscuits
Source: Foods. 2026 Aug 8;15(16):2785. doi: 10.3390/foods15162785 (PMC13512168; doi:10.3390/foods15162785)
Supplement: Supplementary file 1 [file foods-15-02785-s001.zip › foods-4456016-supplementary.pdf]

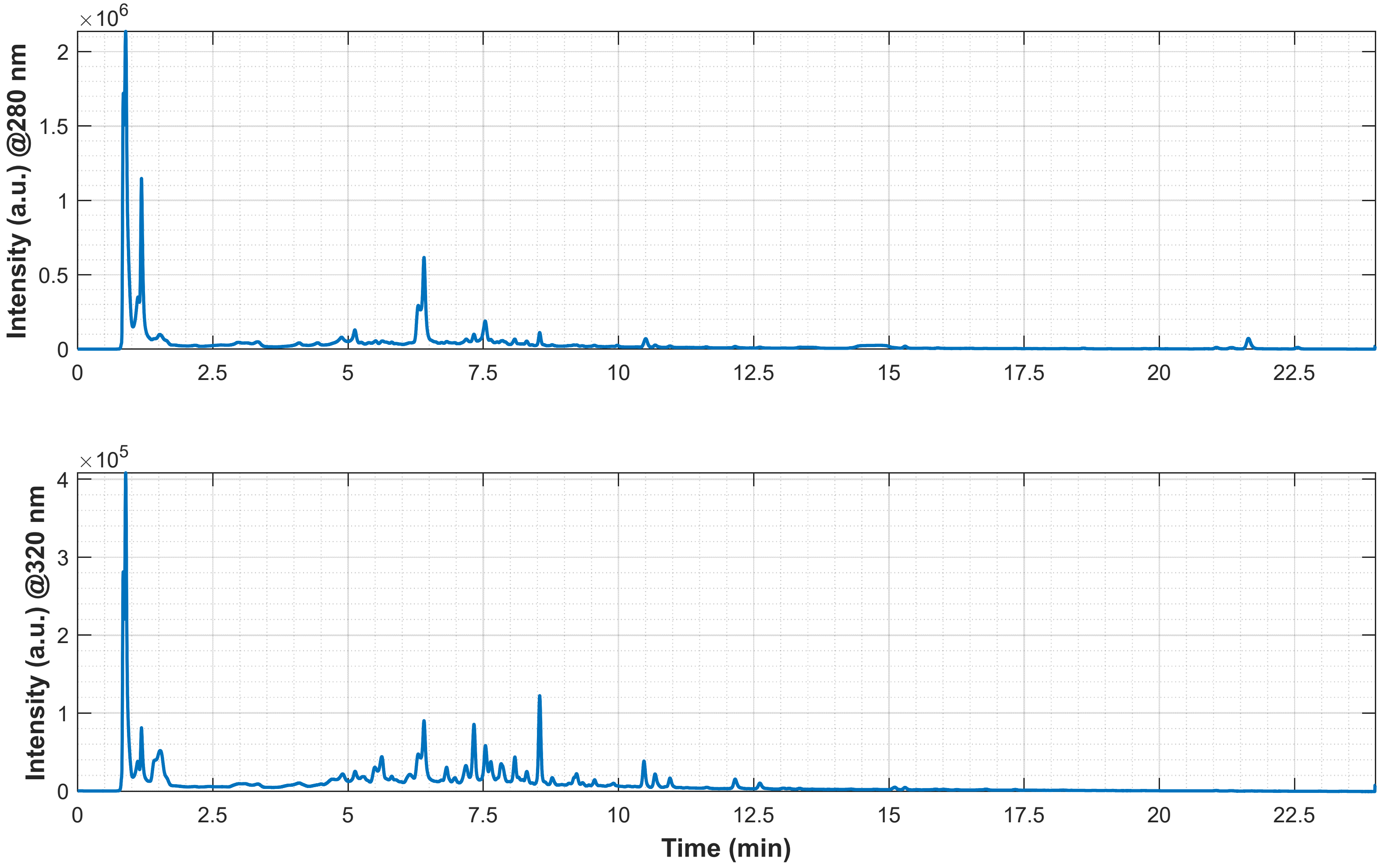

Supplementary Figure S1. LC-PDA chromatograms at 280 and 320 nm of carob flour phenolic extract.
